# Reminiscences of Dr. Mahlon Preston

**Published:** 1895-12

**Authors:** 


### REMINISCENCES OF DR. MAHLON PRESTON.

Many and varied have been the tributes to departed worth that have followed the passing away of Dr. Mahlon Preston, and trusting that yet another may seem neither unseasonable nor inappropriate, we seek to lay it within the same consecrated shrine. He was, without doubt, the pioneer of the homœopathic system of medicine in this locality. Others had preceded him, but confronted, as they were, by a strong tide of prejudice and opposition at every turn, their courage failed.

Dr. Preston was "made of sterner stuff." Believing that "he is thrice armed who hath his quarrel just," and conscientiously convinced of the justice of his cause, no consideration of fortune, fear, or favor could swerve him from his purpose, until, in an incredibly short space of time, his indomitable perseverance and skill as a practitioner had won for him a lucrative position in the midst of some of the most intelligent and influential members of our community.

Among his patrons, whether of high or low degree, his sympathetic and gentlemanly bearing inspired confidence, and the sad faces of those who had come to gaze for the last time upon all that was mortal of Dr. Preston told that they realized the loss, not only of a kind and skillful physician, but a tried and true friend.

In the zenith of his usefulness he hath been summoned to "the higher life," and the question naturally arises, "Why is this?" But the world is full of such wherefores, and turning from them, let us hope that his superior foresight and judgment having chosen his own successor, that fact may contribute largely, as no doubt it will, in aiding the establishment for that successor of the esteem and confidence amongst us which the subject of this notice once enjoyed. L. W. H., NORRISTOWN, PA.

—*Norristown Daily Herald, Monday, November* 11*th*, 1895.

[A word here concerning Dr. Preston's successor, Dr. Charles T. Shinn, referred to in the above article, will hardly be out of place.

Dr. Shinn graduated from Hahnemann Medical College, of Philadelphia, five years ago. He was resident physician of Hahnemann Hospital and is a strict follower of that brilliant man, Samuel Hahnemann, who has given to the world the true healing art. Dr. Shinn does not consider that he can fill Dr. Preston's place, for it takes years of applied study pursued with the remarkable industry and singleness of purpose that distinguished Dr. Preston, to equal his signal ability as a prescriber. But by patience and perseverance he hopes to worthily maintain the reputation for Homœopathy as a reliable system of healing the sick, established by his lamented predecessor.

EDITOR.]

